# Smart biomaterials - regulating cell behavior through signaling molecules

**DOI:** 10.1186/1741-7007-8-59

**Published:** 2010-05-19

**Authors:** Aneta J Mieszawska, David L Kaplan

**Affiliations:** 1 Department of Biomedical Engineering, Tufts University, 4 Colby Street, Medford, MA 02155, USA

## Abstract

Important advances in the field of tissue engineering are arising from increased interest in novel biomaterial designs with bioactive components that directly influence cell behavior. Following the recent work of Mitchell and co-workers published in *BMC Biology*, we review how spatial and temporal control of signaling molecules in a matrix material regulates cellular responses for tissue-specific applications.

See research article http://www.biomedcentral.com/1741-7007/8/57

## Commentary

Tissue engineering is an advanced interdisciplinary field that encompasses the design of artificial implant materials for *in vivo *tissue regeneration where mimicking the natural extracellular matrix (ECM) is often pursued. The natural ECM supports organ and tissue structure and function, and also regulates basic cellular functions like proliferation, growth, migration, differentiation, and survival. These functions are controlled through tissue-specific constituents, such as collagens, laminins, fibronectin or elastins, as well as functional molecules like growth factors or matricellular proteins, among others [1]. Novel biomaterials should allow for the gradual endogenous remodeling of native tissue leading to the replacement of implant material, manufactured to replace a missing biological structure, with fully functional ECM and cells that existed at the implant site prior to damage.

**Figure 1:**
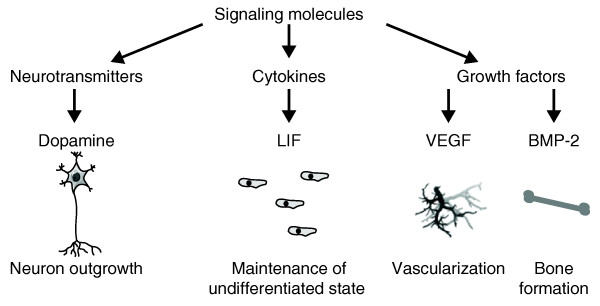
**Examples of signaling molecules modulating cell behavior in bioactive materials for tissue engineering**. The ability of cells to respond to the biomaterial in a controllable manner is a decisive factor in successful disease treatments using engineered implant materials. Signaling molecules involve multifunctional proteins such as growth factors and cytokines, as well as small molecules like neurotransmitters.

The critical point during *in vitro *tissue engineering is to at least partially recreate conditions that mimic the natural ECM environment for particular cell types in order to support their function. Since cell contact with the biomaterial surface significantly influences cell behavior and performance, trends in biomaterial designs lean towards bioactive materials that can modulate and control cell behavior. In recent years, biomaterial designs have focused on the incorporation of signaling molecules into scaffold materials rather than using them in a diffusive or soluble form [1,2]. Among the most studied molecules are multifunctional proteins like growth factors [3-7] or cytokines [8], while there are also reports on the incorporation of small molecules like neurotransmitters [9] into scaffold materials (Figure 1). This review aims at highlighting examples of specific behavior of cell types that was enhanced with signaling molecules tethered to biomaterials.

## Biomaterial-induced vascularization

The development of novel therapeutic approaches that stimulate the body to maintain the natural ability for new blood vessel formation is among challenges of tissue engineering. Endothelial cells play a key role in angiogenesis, through development of new blood vessels from pre-existing vessels. Normally, endothelial cells have a low rate of proliferation but the proliferative capacity is retained and used during such events as wound healing. Fibroblast growth factors are multifunctional proteins involved in processes of proliferation and differentiation of a wide variety of cells and tissues through their stimulatory functions. Sahni and colleagues investigated the natural binding affinity of fibroblast growth factor-2 (FGF-2), also known as basic fibroblast growth factor (bFGF), to fibrin, a fibrous protein formed during wound healing processes, and the ability of FGF-2 to influence proliferation of endothelial cells *in vitro *[7]. Their results showed that fibrin-bound FGF-2 stimulated the growth and increased proliferation of endothelial cells, demonstrating the effect of surface immobilized FGF-2 on the proliferative capacity of endothelial cells. West and colleagues studied the influence of bFGF loaded into hydrogel scaffolds on smooth muscle cell (SMC) behavior [5]. The concentration gradient of bFGF within a gel led to the alignment of SMCs in the direction of increased bFGF concentration. Moreover, the bFGF gradient hydrogels increased SMC proliferation by approximately 41% and migration by approximately 15%, with cell migration observed in the direction of increased bFGF concentration, demonstrating control over cell mobility.

Vascular endothelial growth factor (VEGF) is the other angiogenic protein capable of regulating new blood vessel formation. Influence of VEGF on cell behavior was studied by Zisch *et al. *[6]. VEGF enrichment of polyethylene glycol (PEG)-peptide hydrogels was critical for *in vitro *endothelial cell survival and migration within the gel environment. Also, hydrogel grafting on top of the embryonic chick chorioallontoic membrane led to pronounced VEGF-induced angiogenesis, with strong new blood vessel formation observed at the graft-membrane interface. A rat subcutaneous implant model with VEGF-loaded hydrogels showed complete remodeling of the implant material into native-like, vascularized tissue.

Since locally induced angiogenesis plays an important role in tissue remodeling *in vivo*, *in vitro *strategies such as these may contribute significantly to new biomaterial designs that support therapeutic new vessel growth.

## Modulation of cell proliferation and survival

A major challenge in the tissue engineering field is to provide the environment within the engineered biomaterial to support cellular proliferation and survival, a hallmark of functional tissue. Mesenchymal stem cells (MSCs) are multipotent stem cells able to differentiate into different cell types for regenerative medicine and tissue engineering needs. *In vivo *animal models using MSCs for reconstitution of damaged tissues such as cartilage, bone, muscle, and tendon have shown positive outcomes and promise for human applications. Many clinical approaches for disease treatment using MSCs, like filling large bone defects, will require the implantation of MSCs loaded in or attached to scaffold materials and this could induce an inflammatory response causing large cell losses. Fan and coworkers proposed the incorporation of epidermal growth factor (EGF) into a synthetic polymer matrix material to increase cell survival under such conditions [4]. They found that surface-exposed EGF promoted cell attachment to the matrix material and increased MSC spreading and survival when compared with media containing solubilized EGF. Also, MSCs in contact with surface-tethered EGF displayed a higher resistance to cell death induced by proinflamatory cytokines. These findings provided insight into cell responses to bioactive surfaces and the need to provide local, spatially controlled distribution of signaling molecules on a biomaterial surface. Improving MSC-implant interactions has the potential to enhance cell survival significantly in clinical disease treatments.

## Regeneration of neurons

Nerve growth factor (NGF) covalently immobilized on patterned polydimethyl siloxane (PDMS) surfaces was studied for its potential to induce neuron responses [3]. PDMS is a highly viscoelastic polymer that upon crosslinking and hardening molds the surface patterns it was cast on. Gomez and coworkers [3] investigated the impact of microchannel-patterned PDMS surface on the rate of axon initiation and overall axon length of embryonic hippocampal neurons. The influence of NGF on axon formation was lower (27%) when compared to the influence of PDMS microtopography, which was the decisive factor leading to a 68% increase in axon initiation. Nevertheless, the immobilized NGF played the major role in enhancement of axon length, with an increase of about 10%. The most pronounced effect was observed when the two stimuli, microchannels and tethered NGF, were present simultaneously on the PDMS surface, resulting in a synergistic 25% increase in axon length with enhanced initiation due to surface topography and improved growth from NGF. Another interesting approach to stimulate neurite outgrowth was a bioactive diglycidyl ester polymer bearing dopamine residues [9]. The neurotransmitter activity enhanced neurite outgrowth and induced differentiation in rat pheochromocytoma (PC12) cells, while the diffusive dopamine added to the culture medium had a negligible effect.

## Bone formation *in vitro*

There is a high demand for novel bone replacement materials that can actively support new bone tissue formation in therapeutic treatment of bone loss diseases. There are numerous reports on bone remodeling studies using different scaffolds either in single component or multi-component systems. An example of bone remodeling *in vitro *using bioactive molecules is the report from Birch and colleagues [10] where two different osteogenesis-promoting ligands were immobilized on gold surface. The cell adhesive motif was derived from osteopontin (OPN), a prominent component of mineralized extracellular matrix and the second motif that supported osteoblast differentiation and function was derived from bone morphogenetic protein (BMP)-2, known to influence the development of bone and cartilage. The proposed strategy involved genetic engineering of recombinant fusion proteins where each motif was cloned between the bacterial protein TolAIII, which acted as a scaffold material to support and expose the bioactive OPN and BMP-2 ligands, and Cysteine containing C-terminus tag, used to self-assemble the constructs on gold surfaces. This system provided a self-assembled monolayer (SAM) of fusion protein construct on a gold surface that displayed the ligand motifs in highly reproducible manner. This is a fundamental study that provides an insight into cell behavior in the presence of immobilized bioactive ligands. The OPN construct promoted cellular adhesion and spreading of primary rat osteoblasts through the formation of vinculin adhesion sites. The BMP-2 constructs drove *in vitro *bone formation for over 28 days without the addition of external osteogenic stimuli. The spatial patterning of the BMP-2 ligand led to the local control of osteogenesis and mineralization of the extracellular matrix, offering insight into the spatial control of cell differentiation towards this phenotype.

## Retention of stem cell phenotype

Diffusible leukemia inhibitory factor (LIF) is often used in mouse embryonic stem cell (mESC) culture to retain the cells in an undifferentiated state. Immobilization of cytokines such as LIF and stem cell factor (SCF) to maleic anhydride copolymer thin-film coatings proposed by Alberti and coworkers [8] investigated control over stem cell fate through spatially constrained ligand presentation that provides quantitative control of the signaling ligands at material-cell interfaces. The results showed that mESCs cultured on immobilized LIF surfaces retained pluripotency for at least 2 weeks in culture and, more importantly, that the immobilized LIF ligands were capable of activating the LIF-specific cellular signaling pathways, which support the self-renewal of mESCs *in vitro *in a dose-dependent manner. The significance of these findings comes from the fact that *in vivo *LIF is produced in two isoforms, a diffusible and also matrix-associated form, where the concentration of LIF in both cases is a decisive factor in activation of the LIF-specific signaling pathway. Thus, achievement of precise quantitative control over the polymer-immobilized LIF cytokine may have a great impact in the tissue engineering field. Additionally, ECM coatings on the polymer-LIF surfaces did not affect the ligand accessibility and cell-ligand interaction. This leads to controlling the cell fate through a synergistic effect of ECM of choice and an immobilized cytokine, which broadens the potential applications of this system.

The work discussed in this review represents the emerging area of study aimed at influencing cell behavior through the use of signaling molecules immobilized in biomaterials for tissue engineering needs. These examples follow a trend towards 'smart' bioactive material designs where cell performance can be tuned through the control of dose and spatial distribution of bioactive compounds within tissue scaffolding materials.
